# Symptom improvement in a South Asian patient with Parkinson’s disease treated with immediate- and extended-release carbidopa–levodopa: a case report

**DOI:** 10.1186/s13256-025-05385-x

**Published:** 2025-07-07

**Authors:** Gail Reiner, Michael Skipworth

**Affiliations:** Neurosciences, Movement Disorders Neurology, University of California San Diego, La Jolla, USA

**Keywords:** Parkinson’s, Carbidopa, Levodopa, Movement disorders, South Asian

## Abstract

**Background:**

Little is reported in literature about Parkinson’s disease and treatment responses among South Asians.

**Case presentation:**

Under the supervision of a movement disorders neurologist, an 85-year-old South Asian American man in hospice care was sufficiently regenerated from an adjustment of medications for Parkinson’s disease, such that he was able to shift from being immobile, unable to speak, or safely eat and drink fluids orally, to walking, eating, drinking fluids, and communicating again. His initial symptoms motivated him to seek neurological care in 2012, with his first consultation with a movement disorders neurologist occurring in 2023. After receiving specialty care and adjusting his carbidopa–levodopa from immediate release to a combination short- and long-acting formulation, Rytary, he was discharged from hospice to home health care. He progressed from being almost entirely nonverbal, bed-bound, and solely dependent on artificial nutrition to supportive home-based physical therapy, which facilitated his ability to regain sufficient strength for ambulation, eating and drinking orally, and he can once again use expressive language.

**Conclusion:**

Immediate-release carbidopa–levodopa given three to four times daily remains the most common medication regimen for managing Parkinson’s disease symptoms of tremor and rigidity, with benefits noted in responsive patients for 2 to 3 hours. Extended release and combination formulations of carbidopa–levodopa that combine short- and long-acting medications in one capsule provide symptom relief for responsive patients for 4–6 hours and should be considered when the benefit from immediate-release carbidopa–levodopa is limited. Little has previously been published about the potential for variations in carbidopa–levodopa formulation responsiveness in South Asians, though it has been suggested that Western guidelines for managing “off” periods where carbidopa–levodopa is less effective, may not apply to other ethnicities (Bhidayasiri *et al*. in Expert Rev Neurother 15(11):1285–1297, 2015). As exemplified in this case, greater understanding of non-Caucasian races’ responses to carbidopa–levodopa, extended release, and their combination formulas is needed as is the benefit from subspecialty care within neurology, where the art of medication management is enhanced by experience in movement disorders and where objective tools such as the Movement Disorder Society-Unified Parkinson’s Disease Rating Scale can measure stability, decline, or improvement within each patient pertaining to their activities of daily living, cognitive and motor functions impacted by Parkinson’s disease, as well as monitoring for adverse reactions to medications used for Parkinson’s disease, such as dyskinesias.

## Introduction

The median survival from Parkinson’s disease (PD) diagnosis ranges from 6–22 years and the impact on quality-of-life is progressively more challenging. With the support of movement disorders neurology offering subspecialty care, including expert medication management (that is, the use of newer medications such as Rytary, [a combination short- and long-acting carbidopa–levodopa not available in generic form]), patient and care partner quality-of-life can be significantly facilitated and sometimes dramatically improved. This is the first report of a South Asian American man benefiting from combination short- and long-acting carbidopa–levodopa (CD/LD) rather than instant release CD/LD, exemplifying that people of South Asian descent (and other non-Caucasian races) may respond differently to different forms of levodopa.

## Case presentation

### Background

The patient is an 85-year-old South Asian American man born in Sri Lanka who immigrated to the USA during early adulthood. He received his Bachelor of Science degree in Business, working in business until his non-medical retirement. He lives in a single-dwelling home with his registered nurse wife. They are practicing Buddhists.

The patient’s medical history includes coronary artery disease with a myocardial infarction at age 56 years, hypertension, and type II diabetes. His history is negative for symptoms of prodromal PD: rapid eye movement sleep behavior disorder (RBD), constipation, and hyposmia, with prodromal nonmotor symptoms also negative (that is, sexual dysfunction, depression, or color vision impairment) except for urinary incontinence/nocturia which started at age 67 years.

Patient and family denied pesticide and pollutant exposures pertinent to an increased risk of PD.

The patient developed upper extremity rest tremors in his dominant, right hand at age 72 years, was diagnosed with essential tremor by a general neurologist, and empirically treated with CD/LD 25–100 BID despite beta blockers or primidone being the standard of care for essential tremors. Intermittent bilateral resting hand tremors, with worsening right arm tremors with increased intensity during action began at age 74 years, impairing his ability to sign his name, eat, or drink with his dominant hand, such that he began using his left hand for those purposes; however, he continued his lifelong habit of playing recreational tennis 5 days a week. Magnetic resonance imaging (MRI) was done at age 75 years, which showed mild chronic microvascular changes and generalized cerebral volume loss without acute ischemia.

At age 76 years, memory problems began and tremors progressively worsened in the evening, resulting in a general neurologist increasing his carbidopa–levodopa to three times daily. He became more fatigued after playing tennis with tremors progressing in intensity and distribution to lower extremities and his entire body, triggering perspiration. Neither neuropsychiatric testing nor additional medications were offered, including rescue therapies such as Parcopa, Inbrija, or apomorphine, to reduce the impact of “off” periods [1]. His general neurologist prescribed an additional dose of CD/LD at bedtime, which improved his quality of sleep. No genetic studies or clinical trials were offered. By age 77 years, he had to stop playing tennis due to progressive fatigue. At age 80 years, he developed bradykinesia, with postural instability a year later; however, he has never fallen owing to close supervision and the support of his caregivers.

Concomitant medications for comorbid diagnoses include metoprolol, metformin, amlodipine, atorvastatin, Eliquis, potassium, tamsulosin, and Seroquel as needed for hallucinations, which he has experienced twice, and which stopped due to Seroquel.

The patient’s advanced PD was evident by hypomimia, decreased eye blink rate, hypophonia (rare vocalizations), dysphagia, and an inability to walk. His most pronounced nonmotor symptom was fatigue, with cognition difficult to ascertain owing to almost complete mutism. The patient had three hospitalizations in 2022, the first for urosepsis lasting 1 week. Months later he developed wheezing and was hospitalized for pneumonia for 2 weeks with re-hospitalization within 1 month of that discharge for increased respiratory secretions, profound weakness, and a cumulative 20-pound weight loss. Parkinson’s medications were continued during each hospitalization. During his first hospitalization in 2022, a feeding tube was placed owing to his inability to swallow safely. Once sufficiently stable, he was transferred to a rehabilitation facility for 7 weeks, transitioning at discharge into hospice care due to his inability to progress in rehabilitation and his continuing decline in overall health. He required full care and was bed-bound; however, with the support of caregivers he was able to transfer to a chair for short periods. In May 2023, the patient was hospitalized for 3 weeks owing to respiratory distress as the hospice was unable to provide adequate symptom management, and, after inpatient antibiotic therapy and nutrition support, he was discharged home with the support of home health, which continued for 3 months. He now receives movement disorders neurology medical care through telemedicine with periodic home visits by a physician’s assistant for general medical needs.

After hospitalization in 2023 he was referred to a movement disorders neurologist. Medication changes were made with the addition of combination short- and long-acting CD/LD 48.75–195, which provides one-third immediate time-release beads and two-thirds extended-release beads within one capsule. This extends the medication’s period efficacy a longer period (4–5 hours) than immediate-release carbidopa–levodopa. For 3 weeks, combination short- and long-acting CD/LD was started in the evening dose, while short-acting carbidopa-levodopa continued for three prior doses earlier in the day, with a notable benefit of increased overall alertness. All four doses of short-acting medication were then converted to combination short- and long-acting CD/LD following the initial period. Within weeks of initiating this regime, he maintained extended periods of alertness and achieved the ability to walk with a standing flat form walker or holding his wife’s hand. He can eat again in lesser amounts, which he controls well with the cultural practice of eating with his hand, although the feeding tube remains for fluid and nutrition support. He reads the paper, sits outdoors and greets neighbors, and speaks in short sentences to his family and friends, as well as enjoying watching tennis on television. He has had two hallucinations stopped successfully by Seroquel. When family friends visited, who often perform music, tabla drums, and dancing, he said, “Let’s begin!” The middle-school aged child living in the home noticed that he asks her about her day at school and he enjoys listening to her share information about her classes and sports activities, which fatigue and apathy had made it impossible for him to engage in earlier. In early 2024 he had his first bout of COVID, with mild symptoms lasting 3 weeks. His oral intake declined, and increased nutrition and fluids were required to be delivered through his feeding tube, but he continues his life slightly weaker but still enjoying a positive quality-of-life.

## Discussion

This case highlights the importance of personalizing care of patients with PD with careful consideration for ethnicity, potential implications of genetic variations relevant to treatments under considerations, and an awareness of disparities between populations studied for PD and ethnic minorities. The patient’s dramatic response to an alternative in carbidopa–levodopa formulation concurs with research that validates potential differences in medication response throughout ethnicities since factoring in the likeliness of variable genetic responses to PD medications is highly relevant [2].

The delay in this patient’s diagnosis infers PD may be under-diagnosed in South Asians, and perhaps generally in minority populations. Clinician awareness of symptom variations and proactive use of DaTscans and other diagnostic tools may facilitate earlier diagnosis and treatment. DaTscans have been FDA approved since 2011 to assist in distinguishing essential tremor from PD, though sadly, these scans are not routinely done despite their accuracy (for example, 98–100%) in finding nigrostriatal cell loss in patients with PD [3]. PD remains a clinical diagnosis. In this patient’s case, a DaTscan would have been helpful to distinguishing his essential tremor from PD. Clarifying whether a tremor is essential or a sign of PD is an indication for DaT scan that is commonly reimbursable by insurance including Medicare and Medicaid.

Recommending consultation with a movement disorders neurologist when there is a question about the fit of an initial diagnosis, and/or if disease progression occurs, is appropriate. Every patient should be educated about clinical trials as well as genetic testing for PD.

Little is published on the impact of PD in South Asians during life in their countries of origin or after immigration, despite the diagnosis of 6 million individuals with PD worldwide and PD being the fastest growing neurological problem globally [2]. There is an ongoing need for understanding this disease beyond the most common patients studied—white patients of European descent [4]. Sri Lanka is ranked 168 for total deaths from PD, and the highest prevalence of PD in the USA is among white men when extracted from Medicare records [5]. The likelihood that PD is under-diagnosed in ethnic minorities is genuine and, in this subject’s case, “essential tremor” remained his diagnosis for many years. When newer diagnostic methods are available to confirm the diagnosis of essential tremor, it is important that these methods be offered.

Dementia medications in PD vary significantly among ethnicities, race, and gender [6], and though this patient was showing cognitive changes, he was never offered medication to support optimal cognitive function.

Nocturia, one of the earliest nonmotor symptoms experienced by this patient, was reported in a single study of nonmotor symptoms in Pakistani patients with PD as the most frequent nonmotor symptom of PD [7], so perhaps this case will lead clinicians to consider nocturia as a possible prodromal symptom of PD in South Asian patients.

Quality-of-life outcomes are worse in minority patients with PD [8]. This is evident in this patient’s experience and persistent decline, resulting in hospice admission after years of increasingly intense, frequent, and disabling tremors.

More patients of South Asian descent have been reported to have hypo-responsiveness to carbidopa–levodopa [9]. For patients of Asian descent, South Asians included, region-specific fluctuations of CD/LD wearing-off has been observed to impact the efficacy of proper treatment among these populations [10].Wearing-off of CD/LD is observed through the worsening of symptoms at a current dose and timing of CD/LD, and management of patients with drug regimens is an art requiring systematic follow-up visits and consideration of the patient’s progression of symptoms based on the course of PD, assessed through understanding the length of time and severity they have had PD symptoms, and how these relate to the dose and timing of the CD/LD medication regime. As such, the conversion of this subject’s immediate release to combination IR/SR (Immediate Release/Extended Release) carbidopa–levodopa with higher dosing is relevant in his significant improvement in symptoms, drug responsiveness, and quality-of-life, despite being in the later stages of PD when CD/LD is more likely to have reduced benefits. Since the American Academy of Neurology does not have guidelines for the management of advanced PD, it is critical for clinicians to understand the availability of various formulations of levodopa to best personalize care [11]. Validation of the safety and efficacy of Rytary (IPX203), demonstrating longer motor benefits from this form of CD/LD, should inspire sooner use of this formulation in clinical practice [12, 13]. 

Genetic testing of patients with PD has value for both appreciating the relevance of genes known to be associated with PD as well as those relevant to treatment [14] Catechol-*O*-methyltransferase (COMT) polymorphisms may alter the response to PD drugs since COMT inhibition is required for optimal benefit from carbidopa–levodopa [15]. Early use of COMT inhibitors and/or use of MAO-B inhibitors may potentially reduce the motor fluctuations that are common in PD [16] Unfortunately, genetic testing of patients with PD is not easily accessible, and though it may have a valuable contribution to personalizing treatment, pharmacogenetic testing (also called pharmacogenomic testing)—which typically includes COMT genotyping—is not offered to patients [17]. Recently the groundwork has been laid for evaluating PD gene loci in non-Europeans through a very large genetics study of PD, validating the value of genetic testing for diagnostic and therapeutic purposes in PD [18], and studies specific to South Asians are more easily accomplished with appreciable results in countries that have large genome databases, such as was recently performed in Great Britain where polymorphisms of COMT and other critical pharmacogenes were discovered in two South Asian groups [19]. Recent affirmation of the value of genetic testing has nonetheless acknowledged that the cost and availability of testing is widely variable [20]. An excellent recent review paper on PD therapy concludes with key points that this case exemplifies: early PD diagnosis and communication, treatment initiation timing, and symptomatic pharmacotherapy with consideration for early combination treatment have the greatest chance of optimizing quality-of-life for affected patients [16].

## Conclusion

It is critical to consider the ethno-racial populations studied in research that leads to standards of care for PD, as personalized care is critical when the ethnic background of the patient requiring care differs from the original population studied. Movement disorders neurologists can personalize care and consider genetic testing, as well as a range of CD/LD preparations for which all patients must be considered. Racial and ethnic differences in the identification and management of PD are a known concern and need to be addressed through specific care of patients with PD from racial and ethnic minorities [21] since the quality-of-life for minority patients with PD is also documented to be poorer than those of Caucasian patients https://www.newswise.com/articles/for-people-with-parkinson-s-disease-quality-of-life-linked-to-race-ethnicity.

## Data Availability

Availability of supporting data was available to the clinician author through accessible medical records. Data will not be shared further due to the privacy request of the patient and family.

## References

[1] Masood N, Jimenez-Shahed J. Effective management of “OFF” episodes in Parkinson’s disease: emerging treatment strategies and unmet clinical needs. Neuropsychiatr Dis Treat. 2023;19:247–66. 36721795 10.2147/NDT.S273121 PMC9884436

[2] Armstrong MJ, Okun MS. Diagnosis and treatment of Parkinson disease: a review. JAMA. 2020;323(6):548–60. 32044947 10.1001/jama.2019.22360

[3] Suwijn SR, van Boheemen CJ, de Haan RJ, Tissingh G, Booij J, de Bie RM. The diagnostic accuracy of dopamine transporter SPECT imaging to detect nigrostriatal cell loss in patients with Parkinson’s disease or clinically uncertain parkinsonism: a systematic review. EJNMMI Res. 2015;5:12. 25853018 10.1186/s13550-015-0087-1 PMC4385258

[4] Ben-Joseph A, Marshall CR, Lees AJ, Noyce AJ. Ethnic variation in the manifestation of Parkinson’s disease: a narrative review. J Parkinsons Dis. 2020;10(1):31–45. 31868680 10.3233/JPD-191763 PMC7029316

[5] Wright Willis A, Evanoff BA, Lian M, Criswell SR, Racette BA. Geographic and ethnic variation in Parkinson disease: a population-based study of US Medicare beneficiaries. Neuroepidemiology. 2010;34(3):143–51. 20090375 10.1159/000275491 PMC2865395

[6] Mantri S, Fullard M, Gray SL, Weintraub D, Hubbard RA, Hennessy S, *et al*. Patterns of dementia treatment and frank prescribing errors in older adults with Parkinson disease. JAMA Neurol. 2019;76(1):41–9. 30285047 10.1001/jamaneurol.2018.2820 PMC6382612

[7] Tanveer K, Attique I, Sadiq W, Ahmad A. Non-motor symptoms in patients with Parkinson’s disease: a cross-sectional survey. Cureus. 2018;10(10): e3412. 30538900 10.7759/cureus.3412 PMC6281445

[8] Di Luca DG, Luo S, Liu H, Cohn M, Davis TL, Ramirez-Zamora A, *et al*. Racial and ethnic differences in health-related quality of life for individuals with Parkinson disease across centers of excellence. Neurology. 2023;100(21):e2170–81. 37019661 10.1212/WNL.0000000000207247 PMC10238163

[9] Wijeyekoon R, Suriyakumara V, Gamage R, Fernando T, Jayasuriya A, Amarasinghe D, *et al*. Associations between lifestyle factors and Parkinson’s disease in an urban Sri Lankan clinic study. Int Arch Med. 2017;10:246. 29057010 10.3823/2516 PMC5646647

[10] Bhidayasiri R, Hattori N, Jeon B, Chen RS, Lee MK, Bajwa JA, *et al*. Asian perspectives on the recognition and management of levodopa ‘wearing-off’ in Parkinson’s disease. Expert Rev Neurother. 2015;15(11):1285–97. 26390066 10.1586/14737175.2015.1088783

[11] Livingston C, Monroe-Duprey L. A review of levodopa formulations for the treatment of Parkinson’s disease available in the United States. J Pharm Pract. 2023;37:485–94. 36704966 10.1177/08971900221151194

[12] Espay AJ, Hauser RA, Dhall R, Thakkar S, Cloud L, Zeitlin L, *et al*. Safety and efficacy of IPX203 in Parkinson’s disease: the RISE-PD open-label extension study. Mov Disord. 2024;39(2):428–32. 38111267 10.1002/mds.29685 PMC10922967

[13] Hauser RA, Espay AJ, Ellenbogen AL, Fernandez HH, Isaacson SH, LeWitt PA, *et al*. IPX203 vs immediate-release carbidopa-levodopa for the treatment of motor fluctuations in Parkinson disease: the RISE-PD randomized clinical trial. JAMA Neurol. 2023;80(10):1062–9. 37578800 10.1001/jamaneurol.2023.2679 PMC10425876

[14] Cook L, Schulze J, Verbrugge J, Beck JC, Marder KS, Saunders-Pullman R, *et al*. The commercial genetic testing landscape for Parkinson’s disease. Parkinsonism Relat Disord. 2021;92:107–11. 34696975 10.1016/j.parkreldis.2021.10.001 PMC8633166

[15] Dorszewska J, Prendecki M, Oczkowska A, Rozycka A, Lianeri M, Kozubski W. Polymorphism of the COMT, MAO, DAT, NET and 5-HTT genes, and biogenic amines in Parkinson’s disease. Curr Genomics. 2013;14(8):518–33. 24532984 10.2174/1389202914666131210210241 PMC3924247

[16] Stocchi F, Bravi D, Emmi A, Antonini A. Parkinson disease therapy: current strategies and future research priorities. Nat Rev Neurol. 2024;20(12):695–707. 39496848 10.1038/s41582-024-01034-x

[17] Cacabelos R. Parkinson’s disease: from pathogenesis to pharmacogenomics. Int J Mol Sci. 2017;18(3):551. 28273839 10.3390/ijms18030551 PMC5372567

[18] Kim JJ, Vitale D, Otani DV, Lian MM, Heilbron K, Me Research T, *et al*. Multi-ancestry genome-wide association meta-analysis of Parkinson’s disease. Nat Genet. 2024;56(1):27–36. 38155330 10.1038/s41588-023-01584-8 PMC10786718

[19] Bharti N, Banerjee R, Achalere A, Kasibhatla SM, Joshi R. Genetic diversity of ‘Very Important Pharmacogenes’ in two South-Asian populations. PeerJ. 2021;9: e12294. 34824904 10.7717/peerj.12294 PMC8590392

[20] Saunders-Pullman R, Raymond D, Ortega RA, Shalash A, Gatto E, Salari M, *et al*. International genetic testing and counseling practices for Parkinson’s disease. Mov Disord. 2023;38(8):1527–35. 37310233 10.1002/mds.29442 PMC10461455

[21] Aamodt WW, Willis AW, Dahodwala N. Racial and ethnic disparities in Parkinson disease: a call to action. Neurol Clin Pract. 2023;13(2): e200138. 37064587 10.1212/CPJ.0000000000200138 PMC10101714

